# Spirit of the Foreign Periodicals

**Published:** 1845-07

**Authors:** 


### Periscope;

OR,

### CIRCUMSPECTIVE REVIEW.

" Ore trahit quodcunque potest, atque addit acervo."

### Spirit of the Foreign Periodicals.

### On the Pathological Effects of Alcohol.

Dr. Peters has given us the following account of the necroscopic appearances, which he has observed in the bodies of nearly seventy persons who have died from the excessive use of ardent spirits.

*Head.*—There was always more or less congestion of the scalp and of the membranes of the brain, with considerable serous effusion under the arachnoid; while the substance of the brain was unusually white and firm, as if it had lain in alcohol for an hour or two; the ventricles were in most cases nearly or quite empty.* This peculiar firmness of the cerebral substance was noticed several times, even when decomposition of the rest of the body had made considerable progress:—Typhus fever is the only disease in which we have observed a like firmness.

*Lungs.*—Congestion, amounting sometimes to complete splenisation, of the pulmonary parenchyma was a common appearance. The bronchi were very generally found reddened, and more or less filled with catarrhal secretions.

" We must make particular mention of the infrequency of Phthisis in drunkards: never have we met a tubercular abscess in them, even of the smallest size, while a small number of chalky tubercles was frequently noticed; and cicatrices also were often met with, and were marked by presence of puckering of the surface of the lungs, of solid bodies which were readily felt before the lung was cut into, and when this was done, they were found to consist of lumps or stripes of callous fibrous tissue, around which we rarely discovered a few discrete, grey, crude, small tubercular granulations; in every instance, these appearances were strictly confined to the upper third of the superior lobes, and the rest of the lungs was entirely free from either old or recent tubercular disease."

*Heart.*—This organ was generally flabby, enlarged and dilated, but little, or not at all, thickened: its outer surface was, in most cases, loaded with fat. The blood was often of an unusually fluid consistence. In some cases, where sudden death had been occasioned by the excessive use of ardent spirits, no other ap-

* In a case, related in a recent number of the Illinois Med. and Surg. Journal, the serum in the cerebral ventricles yielded strongly an alcoholic odour: this was so apparent, that it was readily recognised by every member of the Jury.

pearances were found in the body, except the fluid condition of the blood, congestion of the lungs and membranes of the brain, with serous effusion under the arachnoid.

*Stomach.*—As a matter of course, this viscus very generally exhibited some morbid appearances. Sometimes the mucous membrane appeared perfectly white, and somewhat thickened, with distinct, flat, mammellonated elevations of small size. When a quantity of undiluted spirits had been taken shortly before death, the stomach was often found wrinkled or corrugated, as if from the action of a powerful astringent on its inner surface; the mucous membrane exhibiting here and there patches of punctated, star-like or diffused, hæmorrhagic inflammation. "In ten or twelve of the worst cases, in which from three pints to two quarts of liquor had been swallowed within thirty-six or forty-eight hours before death, we found extensive hemorrhagic inflammation of the larger portion of the stomach, with effusion of blood in large patches under the mucous membrane. In several instances in which unknown persons were found in the river, with severe cuts or bruises upon their heads or bodies, we have been enabled to testify positively, from the above appearances of the stomach, and those of the liver and omentum presently to be described, that they had been deep in liquor just before they had fallen into the water, and that in all probability no murder had been committed, as the cuts or bruises would lead one to suspect."

The *liver*, in moderate drinkers, was generally found to be somewhat larger than usual, its texture softened, and its outer surface spotted with patches of fatty infiltration extending two or three lines into the parenchymatous substance, the rest of the viscus retaining its natural colour, and its edges their normal sharpness. In those, who had been more addicted to the abuse of spirits, the liver was still larger, its edges were more obtuse, and the patches of fat on its surface were larger and more numerous. In old drunkards the liver was very large, weighing at least six to eight pounds, often ten to twelve; the edges were very thick and much rounded; the parenchyma almost white with fat, soft, fragile; and the peritoneal covering could be torn off in very large pieces with ease. Granular liver was found in four or five cases only. *The gall-bladder* was always large and filled with bile.

There is no uniform or characteristic change in the *spleen:* occasionally it is rather larger than natural; but, as a general rule, the small size of this viscus contrasted strongly with the very great enlargement of the liver.

The appearance of the *omentum* is very peculiar; it is loaded with an ashy-grey slushy fat. Our attention was called to this sign in Vienna; it is there regarded as so characteristic, that a man is often judged to have been a drunkard, from a glance at the omentum, when the abdomen is first laid open.

The *mesentery* is said to be "always loaded with a thick layer of fat."

The *small bowels* generally contained a large quantity of bile, and their mucous membrane was thickly coated with a very tenacious mucus. In eight or ten of the worst cases, numerous and extensive patches of hemorrhagic inflammation were found, with copious effusion of blood in and beneath the mucous membrane. This may account for the frequency of discharges of blood from the bowels of drunkards.

The whole body of drunkards, with the exception of the brain, generally passes over into decomposition with unusual rapidity.

Dr. Peters closes his interesting paper with the following pathological conclusions on the *modus operandi* and general morbid effects of the immoderate use of spirituous liquors.

"The most important appearances are the fluid and venous condition of the blood, and the great superabundance of fat. According to Steinheimer and Roesch, alcohol acts directly upon the blood, and drunkenness is owing to an alcoholic venous plethora, in which the proportion of hydrogen and carbon in the blood is much increased. The same alteration of the blood occurs in poisoning with narcotic drugs, and the delirium and excitement of the nervous system, produced by them and alcohol, is supposed to be secondary to this change in the quality of the blood. According to Orfila, if a large quantity of alcohol be taken during or shortly after a meal, it coagulates the albuminous portions of the contents of the stomach, and this coagulated albumen passes off almost unchanged into the small intestines. The action of the gastric juice upon other portions of the food is prevented, and they undergo acetous fermentation. A large quantity of pure alcohol also reaches the duodenum, mixes with the bile, which loses its alkalescence, and can no longer be precipitated into insoluble flocculi by the addition of the acid chyme, as is normally the case; in the natural state, this insoluble precipitate from the bile is not re-absorbed, and is cast out with the fæces; but in drunkards no such precipitate ensues, the bile remains fluid and unchanged by the chyme, and a larger portion of it is re-absorbed; hence the bilious difficulties in drunkards and the frequent occurrence of jaundice in them. Large quantities of acid chyme and imperfectly-digested food pass along the small intestines, and even reach the cœcum and colon, where they also undergo acetous fermentation; this is sufficient to account for the dyspeptic difficulties and sour eructations in drunkards. The blood, which returns from the intestines into the vena porta system and liver, is more or less mixed with alcohol, imperfect bile and other impure matters; hence the venous plethora of the vena portæ, and subsequent affections of the liver; as much bile is brought back to the liver, it is, doubtless, re-secreted from it with great rapidity; hence, among other causes, the large quantity of bile which is usually found in the gall-bladder and small intestines.

"The chyle which is absorbed by the lacteals must be very imperfect, and is mixed with more or less altered alcohol; of course the blood which is formed from it is equally imperfect. If alcohol be added to blood which has been drawn from a vein, the blood becomes dark, it loses its normal opacity, becomes transparent, and changes to a cherry-juice-like fluid. With the aid of the microscope, we see the blood-globules gradually losing their red colouring matter, which becomes equally dissolved and diffused through the serum, which assumes the peculiar cherry-red colour; this serum coagulates to the consistence of thick milk, but cannot form solid coagula, and no watery particles separate from it. These appearances agree with those of the blood of topers, which is thick, but fluid: it coagulates very loosely, contains but little fibrine, but much albumen and fat.

"According to Rokitansky, Andral and Engel, the blood in tubercular cachexia is arterial and rich in fibrin; while in the cancerous cachexia and typhous fever, it is more venous, it abounds in albumen, and is deficient in fibrin; hence alcohol would seem to produce a state of the blood opposite to that which occurs in tubercular disease, and is somewhat similar to that which obtains in cancer; therefore, it may prevent the development of the former, and hasten that of the latter."—*The New York Journal of Medicine,* Vol. III. No. 9.

---

### Observations on the Coagulation of the Blood in the Veins in Chronic Cachectic Diseases. By Dr. Bouchut.

About a twelvemonth ago, the author of these observations published, in the French Medical Gazette, two or three valuable papers on the pathology of *Phlegmasia dolens,* as it occurs in lying-in women. It is well-known that an analogous affection is liable to be met with in the course of certain chronic diseases, quite unconnected with the puerperal state; and the object of Dr. Bouchut's present paper is to shew that the cause of this affection is the coagulation of the blood in the veins of the part implicated, and the consequent obstruction to the venous circulation.

He lays down the following position as one of uniform and invariable truth. "In the latter stages of chronic diseases, whenever there suddenly supervenes a deep-seated pain, soon followed by œdema of the cellular tissue, in a limb, we may be assured that the blood is coagulated to a greater or less extent in the veins of the part." This morbid state has been described under various appellations—as *adhesive phlebitis, spontaneous obliteration* of the *veins,* and non-puerperal *phlegmasia dolens.* Hitherto the subject has never been examined in a comprehensive manner; nor has any writer brought together the detached and scattered cases of the disease that have been reported by Andral, Lee, Bouillaud, Cruveilhier and others, with the view of pointing out their analogies and general bearings.

It has been long known that, in the advanced stages of certain cachectic disorders, such as Phthisis and Cancer, the lower extremities are apt to swell and become œdematous. Hunter has alluded to this fact in his work on Inflammation, but without attaching any importance to it. Abernethy and Travers have subsequently noticed it; and after them, Breschet, in his translation of Hodgson's treatise, drew the attention of the profession a little more particularly to the subject, by publishing the particulars of three cases—one of which occurred in a phthisical patient, another in a person labouring under scrofulous disease of the vertebræ, and the third in an invalid who had fallen into a serious adynamic state supervening upon the retrocession of an eruptive fever. One or two cases have been recorded where this lesion of the veins has occurred in the advanced stage of secondary syphilis; but certainly a large majority of the examples of the disease has been observed in connexion with the existence of Cancer and Phthisis. The vessels most frequently affected are the inferior vena cava, the veins of the pelvis, and those of the lower extremities.

Dr. Bouchut makes reference to the following cases of this morbid lesion, which he has recently met with in the hospital practice of M. Rayer and M. Trousseau. 1. Three women, in the advanced stage of phthisis, who were distressed, during the last month of their lives, with a burning pain in their lower extremities, caused by the obliteration of certain of the veins of the leg and thigh. The coagulation of the blood had, on each side, stopped on a level with the iliac veins. 2. A man affected with encephaloid disease of the liver: in this case, the left lower extremity only was œdematous. 3. A woman labouring under calculous Nephritis, and suppuration of the right kidney: both lower limbs were affected. 4. A woman recently recovered from Typhus fever, but who had fallen into an adynamic cachectic condition. She vomited all her food, and every attempt to nourish the system failed. For four days before her death, she suffered most severe pain in the legs, more especially in the hams: the invasion of the pain was speedily followed by œdematous swelling of the parts affected. The cause proved to be an obliteration of the deep-seated veins of both legs, extending up to the middle of the thighs. 5. A boy, who, in consequence of an extensive burn on his back, became affected with hectic fever. One evening he complained of a sharp pain in the left ham; and next morning the entire leg was œdematous, and tender to the touch along the *trajet* of the vessels. The deep-seated femoral and crural veins were found on dissection partially obliterated. 6. A similar case, which occurred in a young girl: both lower limbs were affected with painful œdema, which was discovered to be caused by coagulation of the blood in the deep-seated veins on either side. 7. A woman who had been largely bled in an attack of Pneumonia, and fell into a state of anæmia and excessive weakness in consequence. The left leg became affected with a painful œdema, that was accompanied with considerable distension of the superficial veins. In this case, there was no dissection. 8. A woman affected with disease of the heart: there was obliteration of the deep and superficial veins of the left leg. 9. A man also affected with cardiac disease: the superior vena cava, the deep jugular, the axillary and deep-seated brachial veins, down to the middle of the fore-arm, were found obliterated. In this case, the seat of the obstructed circulation in the venous trunks was readily detected at different points, by the presence of veins that were engorged and reduced to the state of painful cords. (The meaning of this is not very obvious). Upon the surface of the neck and thorax, there was to be seen a network of innumerable veins, here and there agglomerated in the form of vascular masses like an erectile tissue, and in other places like varicose vessels—all serving, no doubt, for the purposes of the collateral circulation. The countenance and upper extremities of this patient exhibited a purple or *cyanosed* appearance, and were also œdematous: there was likewise a large quantity of water in the left pleural sac. The lower half of the body was quite exempt from any serous infiltration. 10. A woman having cancer of the uterus: there was a painful œdema both of the upper and of the lower extremities in this case.

*The Veins most frequently affected.*—The deep-seated veins of the extremities, more especially those of the inferior, are the vessels in which coagulation of the blood, and consequent obstruction of the vascular tubes, are generally met with: occasionally indeed, but this is rare, these phenomena occur in the veins of the neck and head. Abercrombie found, on one occasion, the sinuses of the *dura mater* obliterated in a cachectic child, that died of suppurative fever caused by caries of the internal ear. Bouillaud and Baron have seen the pulmonary arteries and the hepatic veins similarly affected. But, as in these cases, there was (besides the cachectic condition of the system) a lesion of the lungs and liver, it is possible that the coagulation of the blood might have been caused by an inflammation communicated "de proche en proche," and that the fever was not fairly attributable to the existent cachexia.

So much more frequent is the lesion, which we are now considering, in the veins of the lower extremity than in those of any other part, that, out of 51 cases of spontaneous non-puerperal venous obliteration, no fewer than 44 occurred in these veins. The deep-seated veins, it is to be remembered, are much more frequently the seat of the affection than the superficial ones.

The seat of the sanguineous coagulation in veins is almost always at a distance from the place where the lesion, that occasions the cachectic condition of the invalid, happens to be; so that there is evidently no immediate relation, nor result of contiguity, between the venous lesion and the diseased organ. For example, it is not the pulmonary arteries, but the veins of the inferior extremities, that are apt to be affected in Tubercular Phthisis; and in cancer of the stomach it is not the branches of the vena portæ, but the veins either of an arm or of a leg, that usually become the seat of coagulation of their contents. This anatomical fact it is of importance to attend to; as it clearly shews that the alteration of the dark blood, which is liable to occur in the course of certain chronic diseases, is in some degree spontaneous, and is not the result of any irritation transmitted directly from a morbid part to the affected veins; for, if this were so, the coagula would surely extend along the venous trunks in the whole of the intermediate space:—a state of things that has rarely been met with on dissection. Occasionally indeed we meet with cases where this continuity of alteration has been detected; as, for example, in Cancer of the Uterus; when the coagula have been found to extend from the uterine and hypogastric veins to those of the thigh and leg. In such a case, the vascular lesion is clearly the result of a direct irritation, transmitted "de proche en proche."

It is not to be supposed from these remarks that we mean to imply that, in such morbid degenerations of structure as Tubercular Phthisis and Cancer, the veins immediately connected with the diseased tissues are not apt to be more or less completely obstructed by the existence of coagula within their cavities.

Quite the reverse; for this condition is by no means of unfrequent occurrence. All that we contend for is that, when the veins at a distance from the seat of the disease become affected, the morbid condition of the vessels is not continuous from that point, and therefore is not the result of direct irritation.

The *extent* of the coagula, present in venous trunks, varies a good deal in different cases. Sometimes they occupy the veins of only one of the inferior extremities, and quickly pass to those of the other, having extended upwards as high as the lower part of the inferior cava. At other times they commence on both sides at the lower part of the leg, pass up along the thigh, and stop at the fold of the groin without reaching the common iliacs. Occasionally they are found in the axillary, brachial, and anti-brachial veins, and also in those of the neck. It is often truly surprising how the collateral circulation is re-established. It is, as we have already remarked, almost always the large veins of a limb that become obliterated; the obstruction usually being found to cease as we approach the smaller branches.

The *appearance* of the intra-venous coagula, of which we are now treating, most satisfactorily shews that they are not the result of any inflammation of the veins themselves. They (the coagula) do not at all adhere to the parietes of the veins, at least in the early stage of the disease; for there seems no doubt that the presence of the clots may, like that of any foreign substance, give rise to an irritation of the lining membrane of the vessels, and eventually to genuine phlebitis. Here then we have an additional proof that the coagulation of the blood within the vascular tubes is primarily a spontaneous or idiopathic phenomenon. The subsequent adhesion of the parietes of the affected veins and the obliteration of their channels are consecutive processes, the result and effect of the presence of the coagula.

Whenever a venous trunk becomes obliterated, there is always a tendency to œdematous infiltration of the affected limb. When the upper extremities are the seat of this effusion, we not unfrequently find that there is a liability to œdema of the lungs, and occasionally also to hydrothorax, coming on. It is unnecessary to specify the symptoms that accompany the coagulation of the blood within the veins; as they are exactly the same as we observe in ordinary *phlegmasia dolens* of puerperal women. The duration of the symptoms varies according to the time required by Nature to re-establish the circulation by the enlargement of the superficial veins; as these become visibly larger and more distended, so the swelling, pain, and stiffness of the limb are observed to become less and less. The disease seldom or never terminates in suppuration. The œdema may indeed increase so much as to cause the skin to give way at several points, and a gangrenous Erysipelas may supervene; but these phenomena are very different from the formation of matter. Hence it is that we never meet with the symptoms of *purulent infection*—the usual accompaniment of ordinary Phlebitis.

Whether the obstructed veins ever again become pervious—as certainly occurs in some cases of *puerperal phlegmasia dolens*—under the circumstances which we have been considering, has not yet been ascertained; and indeed the speedy death of the patient, in almost every case, forbids us to expect that such an occurrence should take place. The *prognosis* is, as a matter of course, always most unfavourable.

In the *treatment* of this disease, Dr. Bouchut cautions his readers against the use of bleeding, or the application of leeches or blisters to the affected parts—except there is some very unusual peculiarity in the case. All that should be done is to wrap the limb in flannel, relieve the painful feelings by fomentations or soothing liniments, and give opium internally to quiet the general uneasiness. —*Gazette Medicale.*

---

### MM. Becquerel and Rodier on the Changes of the Blood in Disease.

In our last Number (p. 582), we gave a short notice of these gentlemen's researches on the composition of the Blood, wherein they shewed that a very marked change exists in the relative quantities of some of its constituent parts in the male and female. If their conclusions on this point be correct, it is most necessary that hematologists be well acquainted with the fact, as it cannot fail to have considerable influence in medical reasonings on several pathological subjects of high interest. The following observations on the changes, which the circulating fluid undergoes in the course of certain diseases, are worthy of notice in the present day, when so much attention is justly paid to the question of humoral modifications. It may be asserted as a general fact, that the effect of bloodletting on the composition of the blood itself is chiefly to diminish the proportion of its red globules, without affecting in any considerable degree that of its fibrine, or of the solid contents of the serum: the decrease of these elements, when it occurs, seems to be a result of the decrease of the morbid action, and not of the quantity of blood artificially withdrawn. The experiments of MM. Becquerel and Rodier amply confirm the accuracy of MM. Andral and Gavarret's observations on this point. It is of some importance to keep this fact in remembrance, when we seek to follow out the interesting and highly useful subject of Therapeutic medicine.

Let us first briefly consider the pathology of the Fibrine. The proportion of this element is invariably increased above the normal standard in all genuine Phlegmasiæ. This, in short, is the fundamental and essential character of all inflammatory diseases; without it, an inflammation cannot exist: *voila un fait incontestable et acquis à la science.* This increase in the quantity of the fibrine is invariably found to exist, whatever may be the composition of the blood in other respects, and the proportion of its other constituents. For example, the proportion of the red globules may be less than in health in some inflammations: nevertheless, that of the fibrine is always considerably above the natural standard. But, besides this modification of the circulating fluid, there is very generally, at the same time, another that deserves to be attended to; viz: an increase in the proportion of the Cholesterine, and a diminution of the Albumen, in this class of diseases. In what manner are we to account for these phenomena? The subject is indeed a difficult one; but we must not shrink from the attempt. Let us first consider the latter of these two facts: how shall we explain it? Perhaps the following suggestion may be worthy of notice. Recent researches in organic chemistry have tended to shew that the two elements—fibrine and albumen—are in fact the same substance only somewhat modified; at least, that the former of these elements is derived from the latter. This being the case, may we not suppose that the increase in the quantity of the fibrine may be owing to a transformation of a certain portion of the albumen into it?—a transformation that might be readily effected, seeing that the composition of the two bodies is very nearly alike. A question indeed will here naturally occur to every one:—is the total amount of the increased quantity of the fibrine, and of the diminished quantity of the albumen, in an inflammatory disease equal to that of these two elements in a state of health? MM. Becquerel and Rodier say that it is so; and from this very circumstance, they derive an argument in favour of the hypothesis we have alluded to.

But what shall we now say as to the increase in the quantity of the Cholesterine? This increase is very considerable; for the quantity of this element in certain Phlegmasiæ has been found, by our two authors, to be nearly double of that which is present in the normal condition of the blood. They have proposed the following explanation. In no diseases, is the diet of a patient kept so low and abstemious as in all active inflammations. The consequence of this is that the biliary secretion becomes much diminished or even arrested; and the result of such a diminution or arrest is, that a greater amount of the constituents of the bile remains uneliminated and unchanged in the blood.

Before quitting this part of our subject, there are two points that may be briefly noticed with advantage. The following extracts will probably best serve to bring them under the notice of the reader.

"In the Pyrexiæ, the deviation in the composition of the blood from the standard of health is by no means either so constant or so uniform as in the class of the Phlegmasiæ. The proportion of the fibrine *may be* diminished, and even its physical properties *may be*, and most probably are, very sensibly altered in adynamic and malignant fevers. Nevertheless these are neither universal nor necessary phenomena; and we can attach the less importance to the former of these changes, as we find that the normal proportion of this element is sometimes observed to be notably diminished, without there being any marked deterioration of the health of the individual. As yet, it must be confessed that we do not at all understand the conditions of the system, that are apt to induce the hæmatic alteration in question. It seems, indeed, highly probable that, whenever there exists a tendency to passive hæmorrhages—as is frequently the case during the course of grave fevers—there should be a diminution in the quantity, or a modification of the properties, of the fibrine. In Purpura and Scorbutus also, there is good reason to believe that the blood is deficient in its normal proportion of fibrine. In Typhoid fevers, the proportion of the red globules is generally found to be below the standard of health, and also that of the albumen in the serum to be diminished. On the whole, however, it must be confessed that the blood in this family of the Pyrexiæ does not exhibit any decided or constant changes that can be viewed as at all characteristic of the morbid condition."

*Whenever any secretion is either much diminished or entirely interrupted, it often happens that certain of the elements, which enter into its composition, are found to be present in the blood.* This proposition is well illustrated by the following two grand facts.

A. Professors Provost and Dumas were the first to prove that, when the urinary excretion is prevented by tying the ureters, Urea, which is naturally eliminated by the kidneys, is found in the blood of the animal. It is more than probable that such an occurrence takes place in the human body, whenever the secretion of the urine is wholly or partially suppressed. Of late years it has been very generally asserted that, in the *morbus Brightii,* there is a sensible diminution of the normal proportion of the Urea in the urine, and the discoverable existence of it to a certain extent in the blood. But M. Becquerel asserts that the first of these positions is not strictly correct;* and he also questions the accuracy of the second, in consequence of certain experiments performed by himself and M. Quevenne, the head chemist of La Charité hospital, in which they could not detect any portion of Urea in the blood of patients affected with the disease.

B. Whenever the secretion of the Bile is much diminished, the quantity of Cholesterine in the blood is immediately increased; its degree of accumulation augmenting, as the amount of the secretion becomes less and less. Now such an occurrence very generally takes place under the influence of low diet, especially if the bowels be constipated at the same time. In *jaundice* the accumulation is still more considerable, so that in some cases the quantity of Cholesterine present in the blood is three or four times greater than in a state of health.

*The proportion of the Albumen in the Serum decreases to a considerable extent in three morbid states; viz. in the morbus Brightii, in certain diseases of the heart accompanied with dropsy, and in grave puerperal Fevers.*

---

* Vide *Semeiotique des Urines,* par A. Becquerel.

This proposition is not to be regarded as yet absolutely and definitively proved, although it seems to be highly probable from the results of experiments hitherto made. Medical men are only beginning to direct their attention to the examination of the fluids in disease, and we therefore cannot expect complete uniformity in the conclusions to which different observers have arrived. All that can be done at present is to stimulate enquiry, by recording the facts that have been noticed, and suggesting hints for the further prosecution of the subject.

In no disease is the diminution of the quantity of the *red globules* more conspicuous—at least, in most cases—than in Chlorosis: there is also a very sensible decrease in the normal proportion of the *iron* in the blood of persons affected with this disease. MM. Becquerel and Rodier are of opinion that this diminution of the former element is rather one of the *effects*, than the proximate *cause*, of the malady, as many medical men seem to suppose. "Our observations," they remark, "lead us to believe that Chlorosis is a disease characterised by a certain number of disorders, of which the most constant and conspicuous, although not the only one, is a decrease in the quantity of the red globules. It is so true that this modification of the blood is only a consequence of the disease, that we rarely observe the decrease to be always to the same extent, under similar circumstances, or even in the same case at different times: it may be inconsiderable at first, and only become more and more decided as the malady continues. The decrease in the amount of the red globules is by no means proportionate to the intensity of the disorders present; and it may be that Chlorosis sometimes exists, without there being any decided decrease in the proportion of this element."

That Chlorosis is not merely a form of *anæmia* is shown, our authors think, by the following circumstance.

"In chlorotic patients there may exist a veritable plethora, or surcharge of the vascular system; whereas no such thing is ever met with in that condition of the system that is induced by profuse hæmorrhage. Now, it is the existence of this very plethora which will best account for the occurrence of certain symptoms of the disease, and explain how it comes to pass that bloodletting is occasionally required for its successful treatment."

We have already said that the proportion of the Fibrine is usually greater in this disease than in healthy blood. This is a curious fact, and one that is not generally known. Many medical men are apt to imagine that it is only in inflammatory conditions of the system that such a state of the blood exists; but such is not the case; for it has been distinctly proved, by the observations of recent experimenters, that the proportion of the Fibrine of the blood is pretty generally higher than the normal standard, not only in Pregnancy, but also in Chlorosis and Consumption.

---

### Condition of the Blood in Tubercular Phthisis.

According to the researches of MM. Andral and Gavarret, there are two modifications of the blood in this too common disease. The *first* is a diminution in the proportion of the red globules—a state of the circulating fluid "that coincides with the commencement of Phthisis, and perhaps precedes it, and which is found to exist in all cases where, from whatsoever cause, the vital powers of the system have lost their energy."—*Traité d'Hematologie, p.* 171.

The *second* modification consists in an increase in the proportion of the Fibrine, —an increase which is only produced, when an inflammatory action has been established around the organic or tubercular deposit.

The former change precedes the development of the tubercles, and persists as long as this continues to take place; while the latter is only the effect of a complicating malady that is induced by their presence in the pulmonary tissue.

MM. Becquerel and Rodier remark, in reference to their observations on the blood in phthisical patients, that "in those in whom there is either a softening of the tubercles, or a pleuritic effusion as a complication, the blood is found to be altered, as if the individual were affected with an active inflammation. The examination of the blood, drawn at a first venæsection, and at an early period of the disease, usually exhibits a diminished density of the blood and of the serum; a diminution in the quantity of the *red globules*, as in every phlegmasia and acute disease; a diminution in that of the *albumen*, less decided however than we find to exist in grave phlegmasiæ; an increase in the quantity of the *fibrine*, of the phosphorated *fatty matter*, and also of the *cholesterine*. Such being the case, we may assert with truth that phthisical patients are placed—as regards re-action, fever, diet, &c.—very nearly in the same condition as persons labouring under an inflammatory disease. The only difference between them, which we have discovered, is a remarkable one; it is the diminution of the animal *soap*—a diminution more considerable than in any other malady."

It may be worthy of notice here, that Felix Boudet recently announced to the Academy of Medicine that he had detected a notable quantity of fatty matter, and particularly of cholesterine, in tuberculous deposits, as well as in the livers of phthisical patients; and that he had been led to believe that there must be a considerable increase of fatty matter in the blood itself of such persons. The researches of MM. Becquerel and Rodier do not however give any sanction to this opinion.—*Gazette Medicale.*

---

### THE FATTY LIVER IN PHTHISICAL PATIENTS.

"My brother," says M. Boudet, "recently sent me a portion of a fatty liver, which was so light that it floated in water, for the purpose of chemical examination. Treated with æther, it parted with nearly one-third of its weight; the loss being of a soft fatty matter, that consisted of oleine, margarine and cholesterine. The chemical composition of the liver might be stated thus:—

| | |
|---|---|
| Water . . . . . . . . . . . | 55,15. |
| Animal matter dried at 100° . . . . . . | 13,32. |
| Fat formed of oleine and margarine, being slightly acid. | 30,20. |
| Cholesterine . . . . . . . . . . | 1,33. |
| | 100,00 |

A healthy liver was found to consist of

| | |
|---|---|
| Water . . . . . . . . . . . . | 76,39 |
| Animal matter dried at 100° . . . . . . | 21,00 |
| Saponifiable fatty matters . . . . . . | 1,60 |
| Extractive matters soluble in æther . . . . . | 0,84 |
| Cholesterine . . . . . . . . . . . | 0,17 |
| | 100,00 |

By comparing these results, we perceive the greatness of the change that had taken place in the chemical composition of the liver, by the morbid degeneration of its parenchymatous substance. The proportion of saponifiable fatty matter had increased 18 times, and that of the Cholesterine at least 8 times; while the quantity of animal matter had fallen to nearly one-half.

It follows from this observation that, under the influence of Pulmonary Phthisis, while the Cholesterine is accumulating in the pulmonary tuberculous deposits,* it is present also in an unusual quantity in the liver, in which organ it is associated with an immense quantity of matter. This curious circumstance may possibly be explained thus: the oleine, the margarine and especially the cholesterine (which in 100 parts contains 97 of Carbon and Hydrogen), are substances that abound in combustible materials, and, as these materials must consequently require a considerable proportion of oxygen for the purpose of combustion, this can only take place when the respiration is active and complete. When this function, therefore, becomes embarrassed and imperfectly performed, the combustion is only partially effected, and consequently the combustible materials accumulate in the blood, and are by it deposited in the parenchymatous tissue of certain organs."—*Encyclographie des Sciences Medicales.*

---

### M. Rayer on Phthisis in Man and in the Lower Animals.

In the number of the Medico-Chirurgical Review for October 1842, we gave the most important conclusions which this truly eminent physician had drawn from his researches upon the comparative study of pulmonary Phthisis in man and in animals. He has not failed to prosecute his labours in this interesting field of pathological enquiry; some of the results of his later labours we now submit to our readers' attention.

I. In the lungs of ruminant animals, and more especially of the sheep, independently of the granulations that are usually recognised as tuberculous, we not unfrequently find other granular points of the same size and outward appearance as the former, and from which a small worm (*strongylus filaria*) may be extracted with the point of a needle. I have met, says M. Rayer, with these *verminous granulations* more frequently in the months of June and July than at any other time of the year. Analogous granulations have been found by Redi in the lungs of a fox. In horses, that have laboured under chronic glanders, another sort of granulations is occasionally observed. These are of a reddish hue at first, and are afterwards found to contain a drop of pus, and subsequently a yellow looking matter that is sometimes associated with a minute deposit of Carbonate and Phosphate of lime.

II. It is generally supposed that the *cretaceous* or *calcareous concretions*, that are not unfrequently found in the tissue of the lungs, are the result of the last modification of tuberculous deposit. This is unquestionably a mistake. The minute miliary deposits of pus, which are apt to form in the lungs and lymphatic glands, are much more frequently the cause of these concretions than tubercles. In glandered horses, and even in old animals that have never been either glandered or tuberculous, I have frequently found these concretions, not only in the lungs, but also in the submaxillary and such interbronchial lymphatic glands, as have once been the seat of inflammation. The appearance of the lungs in old men and women confirms this view of the case; for, in them, we often find points of cretaceous deposit in small indurated portions of these organs; they are usually of a blackish colour, and resist the edge of the scalpel. The circumstance therefore that these concretions are so much more common in the lungs of aged persons who, it is to be remembered, are rarely affected with genuine phthisis, and also in the lungs of old animals, such as the dog and the horse—animals that are scarcely ever the victims of this disease, but are unusually subject to

---

* The researches of M. Boudet lead him to the conclusion that the proportion of this principle (Cholesterine) is ten times as great in tuberculous matter, as in healthy pulmonary tissue.

pneumonic attacks—affords pretty strong proof that they are not the result of tuberculous disease.

III. In the lower animals, as well as in man, the left lung is more frequently diseased than the right; but there is this remarkable comparative difference as respects the ordinary seat of tuberculous deposits:—in many of the former it is the middle and often the lower part of the lung, in which they frequently exist; whereas, in the latter, it is pretty uniformly the upper lobe.

IV. Some years ago, it was the custom to exaggerate very greatly the influence of pneumonic inflammation in stimulating the production of tuberculous disease; in the present day, there is a tendency to run to the opposite extreme, and assert that inflammation of the pulmonary tissue is a rare occurrence in Phthisis. This assertion is unquestionably not correct, as respects not only human beings, but the lower animals also. In the cow, for example, when affected with Phthisis, we may sometimes follow the various degrees or stages of inflammation—from the primary red engorgement of the pulmonary parenchyma to its gray induration—around, and in the neighbourhood of, the deposits of tuberculous matter. In the Quadrumana also and in Birds, the tuberculous masses are often observed to be surrounded with a sort of shell, of a reddish-gray colour, formed by the indurated pulmonary tissue.

V. In *ruminant* animals, it is very common to find, along with tubercles, vesicular worms (Echinococci and Cystocerci) in the diseased lungs: such an occurrence has scarcely ever been observed in the human subject. From this occasional coincidence of Tubercles and Hydatids, it has been conjectured by some speculative pathologists that the former morbid formation is the result of the degeneration of the latter—a doctrine that the very general absence of any of these vesicular worms in the lungs of phthisical patients, not only in man, but also in the Quadrumana and Carnivora, may be considered abundantly to disprove. It is equally a mistake to imagine, as some have done, that there is any relation between the formation of tubercles in the domestic pig and the development of the Cystocerci, with which this animal is much more frequently affected. In truth, there is no more connection between these two lesions than between the formation of tubercles, and the appearance of the *Strongyli,* which are occasionally found in the bronchi of this animal.

VI. Chronic Pleurisy is very commonly associated with tubercular disease of the lungs, not only in the human subject, but also in the lower animals. In the latter, more frequently than in the former, deposits of tuberculous matter are often found in the pseudo-membranous adhesions between the costal and pulmonary pleuræ. In birds, the air-sacs are sometimes so much infiltrated with grains of a friable, yellow-coloured substance, that they form sorts of hard and hollow shells; the internal surface of which—as well as of other cavities to which the air has free access, as for example the bones—is sometimes lined with a veritable *mouldiness,* such as is commonly found on decayed animal matters. This complication must, as a matter of course, greatly add to the embarrassment of the breathing. Since the attention of the pathologist was first directed to this phenomenon of vegetable fungi growing on the morbid tissues of a living animal, by M. Rayer in 1815, it has been repeatedly observed by others in different species of birds.

M. Rayer has met with only one similar instance in the human subject. It occurred in a phthisical patient in whom the cavity of the eighth pleura,—which was filled with air and a small quantity of fluid at the same time—communicated by an ulcerated opening with the bronchi. The pleura, on the anterior part of the chest, was lined with a false membrane, which exhibited, at two points, spots of distinct and veritable *mouldiness.* An analogous phenomenon is occasionally observed in the mouth of new-born infants affected with the *muguet,* on the crusts of *tinea favosa,* and on the surface of gangrened parts.

VII. The relation of certain morbid changes of the liver with the existence of

Pulmonary Phthisis has attracted the attention of all recent pathologists. There is very considerable difference between man and the lower animals in respect of this morbid coincidence. In the former, the liver is rarely infiltrated with tuberculous matter, even when the lungs are crammed with it. In sheep, however, this coincidence is tolerably frequent; and, in the quadrumana, it is still more so; in them, the surface of the liver often exhibits numerous tubercles in various stages of development. In birds, also, the liver and lungs are often the seat of tuberculous deposits at the same time.

The *fatty degeneration* of the former viscus is much less frequent in the lower animals, when affected with phthisical disease, than it is in the human subject. Certain writers have carried their theoretical views further than facts warrant, when they have asserted that, in order to produce the 'foi gras' in geese, the birds are made to fall *into a sort of marasmus*, by enclosing them in dark cages, and feeding them with a particular sort of aliment. I have repeatedly examined Strasbourg geese, in which the liver had become enormously enlarged and fatty; but in all of them the lungs were perfectly sound, and the whole body was loaded with fat. The blood of these birds sometimes presents a milky appearance, in consequence of the quantity of fatty matter contained in it—an appearance which has never been noticed in the blood of phthisical patients.

VIII. In children, affected with Phthisis, the serous membranes, and especially the peritoneum, often exhibit tubercular granulations on their surface. In the Monkey tribe, the abdominal viscera are not unfrequently found to be glued together into one mass by adhesions in every direction, and these adhesions to be infiltrated with an innumerable quantity of miliary tubercles. The same morbid appearance is not unfrequently observed in Birds.—*Archives de Medicine Comparée. Encyclographie des Sciences Medicales.*

### French Report on Vaccination.

This report was read by M. Serres at the seances of the Royal Academy of Sciences, on the 21st of February and 3rd of March. The following are the general conclusions with which it terminates.

1. The preservative power of Vaccination is absolute in a large majority of individuals, and temporary only in a small number. Even in the latter case, it is almost absolute up to the period of adolescence.

2. Small-pox rarely attacks vaccinated persons before the 10th or 12th year of their age. From this period to the 30th or 35th year of life, the liability to variolous infection is greatest.

3. Besides its preservative virtue, Vaccination introduces into the organisation a property or power which attenuates (so to speak) the symptoms of small-pox, shortens their duration, and very considerably diminishes their severity and danger.

4. Cow-pox lymph, taken directly from the animal, gives to the local phenomena of Vaccination a more decided intensity, and its effects are more certain than when old virus has been used. It would seem, however, that, in the course of a few weeks after its transmission to the human system, this local intensity no longer exists.

5. The preservative power of cow-pox does not appear to be intimately connected with the intensity of the symptoms induced by Vaccination: nevertheless, in order that the properties of the lymph be duly preserved, it will be prudent to regenerate it as frequently as possible.

6. Among the means that have been proposed to effect this regeneration, the only one deserving of confidence is that of deriving the vaccine lymph occasionally from its parent source.

7. Re-vaccination is the only sure test that we have to enable us to distinguish such vaccinated persons as are decidedly proof against the infection of small-pox, from those that are only partially or imperfectly so.

8. The result of re-vaccination does not afford a certain proof that those vaccinated persons, in whom it takes effect, were destined to contract small-pox; but only a high probability, that it is especially among such individuals that the disease is likely to occur.

9. As a general rule, re-vaccination should be practised at, and from, the 14th year of life. If small-pox, however, be epidemic, it will be prudent to anticipate this age, and re-vaccinate from the 8th or 9th year.

Among the prefatory observations of the report, allusion is made to the circumstance that Vaccination will sometimes *take* most perfectly in persons who have had small-pox. M. Moreau, the distinguished accoucheur of Paris, assures us that he has vaccinated himself with effect three different times, although he had the small-pox in his youth.

An official document, published by the Government of Wurtemburg, and wherein it is set forth that, out of 1677 cases of small-pox which occurred from the year 1831 to 1836, no fewer than 1055 were in vaccinated persons, contributed very materially to encourage the performance of re-vaccination in most parts of Germany.

The reports, too, of various epidemics of small-pox of late years in this country (France) clearly shew that the proportion of vaccinated persons, who have become affected with the disease, is more than one-third of the entire number attacked. The importance, or rather the necessity, of re-vaccination is therefore strikingly apparent. We have good reason for believing not only that multitudes have been preserved from variolous contagion by having recourse to this measure, but also that the disease has thus been actually arrested in its progress by, as it were, a barrier which it could not overleap.

The effects of re-vaccination in the Prussian army, since the year 1833, have almost completely extirpated small-pox from its ranks. In the Kingdom of Wurtemburg also, it has been found that out of 14,384 soldiers and 19,864 civilians, who were re-vaccinated, only one case of Varioloid has occurred among the former, and only three among the latter, during a period of five years.

Since the year 1830, when the practice of re-vaccination became general, no epidemic of small-pox has been experienced in that kingdom. The good effects of this practice have been not less striking in some of the Italian states.—*Comptes Rendus.*

---

### ON THE STATE OF THE FIBRINE OF THE BLOOD IN INFLAMMATIONS.

To Signor Polli, the author of a clever work on the blood, the following observations are due.

The Fibrine, it is well-known, is in a fluid state during life, and remains so for a short time after the blood has been drawn from the body. All blood coagulates more or less distinctly, before it passes into a state of putrefaction. Those, who have spoken of the blood being in such a dissolved condition as not to exhibit any appearance of coagulation before putrefaction, have been either hasty or inaccurate in their observations.

"I have oftener than once," says Dr. Polli, "caused the coagulation of blood, taken in a fluid state from a body that had been dead for 36 or 48 hours; and I may here remark, *en passant,* that cadaveric rigidity and resolution appear to depend, in all cases, on the fibrinous clot being either formed, or retarded, or redissolved in the capillary vessels of the subject."

The process of Inflammation gives rise to three principal modifications of the

*fibrine* of the blood—viz. an increase of the quantity, a diminished tendency to coagulate, and, lastly, a molecular rarefaction. M. Polli—following the example of chemical writers in their nomenclature of bodies that are isomeric, or, in other words, which retain the same composition although their properties are different—proposes to call the second of these modifications *bradifibrine*, and the third *parafibrine*. The increased quantity of the fibrine, and its diminished tendency to coagulation, being two effects of inflammation in the blood that are well known, we shall confine our present remarks entirely to the phenomena of its molecular rarefaction, or in other words to the *parafibrine*.

It is a well-known fact that a liquid, which holds dissolved a solid body specifically heavier than itself, acquires an increased density. Now, the contained fibrine—which is specifically heavier than serum, since it falls to the bottom of the vessel in which the blood is contained—when it exists in the liquid state in the blood, diminishes, under the influence of active inflammation, the density of this fluid, so that the serum in which it exists is specifically lighter than the same serum when deprived of its fibrine. In other words, the fibrine, in this peculiar state of fluidity, produces such a rarefaction of the principles of the blood, more particularly of its serum, that it renders it specifically lighter than when it is defibrinated. The following experiment, one of many, may be briefly quoted here: A woman, in the eighth month of pregnancy, was seized with severe pneumonia, for which she was bled several times. The blood first drawn indicated, at the moment of its detraction, 6,1 on the areometer; and 6,3 after it had been defibrinated. At the sixth bleeding, it indicated 5,4, both while it was flowing from the vein and after the fibrine had separated. At the eighth and last operation, it had fallen to 4,5 at first, and 4,6 after the blood had lost its fibrine.

We may therefore conclude that the fibrine of the blood may, under the influence of disease, lose its ordinary density, or become rarefied; that, thus modified, it may impart its tenuity to the sanguineous mass in which it exists; and lastly that, in this state, it is at least more rarefied or less dense than the albumen of the serum. But such a conclusion must not be adopted as a general or invariable truth; for the fibrine of the blood, in a person affected with an inflammatory disease, does not exhibit either uniformly, or in all parts of the body, the characters we have mentioned. The *parafibrine* is only one of the modes in which the fibrine may exist in consequence of the act of inflammation. While in the first degree of this morbid process there is simply an augmentation in the quantity of this substance, and, in the second or more severe degree, there is produced the *bradifibrine*, it is in the most severe or advanced of all—at least, (and this is an important reservation) in the most advanced degree of those cases that are susceptible of complete resolution, and consequently below the mark of suppuration and gangrene—that the formation of *parafibrine* takes place.

Now let us briefly notice some of the characters, by which Parafibrine may be distinguished.

Usually it coagulates very slowly; and its coagulation takes place by such fine, loose, and transparent filaments that they are scarcely visible to the naked eye, and, when regarded with the serosity that is retained, they constitute a mass whose appearance is rather gelatinous than fibrinous and coriaceous. The delicate web, which this sort of fibrine forms in the process of solidification, may be compared to the cellular web that gives a sort of consistence to the white of an egg, or to the vitreous humor of the eye: only it is rather more consistent.... The serum, contained in the bullæ of a blister or of a burn, is rich in *parafibrine*. To separate it, all that we need do is to put such serum aside for a short time. If the fluid be then decanted, we find a gelatiniform coagulum, which, at the end of twelve or twenty-four hours, becomes converted into a fibrous membranule that falls to the bottom.

When, along with the parafibrine, the blood contains a certain quantity of ordinary fibrine, or of *bradifibrine,* the coagulation of these substances takes place, not only at different times, but also with a peculiar appearance in each case. Thus we observe, at first, filaments of fibrine that are white, opaque, and like a web that has large meshes, and has a somewhat radiated appearance. Among these filaments, the *parafibrine* is subsequently deposited, gelatinous and more or less transparent.

These remarks may tend to suggest some explanation of an important pathological phenomenon, viz. the fibrinous or plastic and the sero-fibrinous transudations, which seem to constitute the issue or termination of inflammatory action in membranous structures on most occasions. Hitherto it has been difficult to explain how these depositions or extravasations took place; but if we bear in mind that, under the influence of inflammatory disease, the fibrine—or, at least, a certain part of it—becomes of a thinner consistence than the serum itself, we may readily conceive, *a*, that it may transude through the parietes of the blood-vessels; *b*, that, when once escaped from the circulatory system, it may collect together, expanding perhaps into the false membranes, or falling down to the bottom of an effused fluid; and lastly, that these various mutations, as well as the formation of the *parafibrine,* take place only under the influence of a certain degree or severity of inflammatory action, as we have already mentioned. This is higher than that required for the production of the *bradifibrine:* at least, several considerations lead us to believe so. For example: 1, the *parafibrine* is found in the blood drawn at the first bleedings; but very rarely in that which is drawn during the progress of a severe phlegmasia. It is therefore an accompaniment of the disease when this is most violent, disappearing before the formation of the *bradifibrine,* and for some time previous to the simple augmentation of the normal fibrine. 2. The *parafibrine* may be obtained from a vesicated surface, not when the subjacent dermis is pale, but when it is red, and about to secrete pus shortly. Serosity, parafibrine, and purulent matter—such is the regular order of the secretions successively exhaled from the skin, in proportion as the existent irritation becomes more active and intense.—*Annali Universali di Medicina. Gazette Medicale.*

---

### CRANIO-MALACIA, OR SOFTENING OF THE BONES OF THE HEAD.

About three years ago, Dr. Widtmann was called to a child, nine months old, who had died suddenly and most unexpectedly on its mother's lap. The attendants drew his attention to the state of the occiput: it was quite devoid of all hair, had a bluish aspect, and was so soft and unresisting to the pressure of the finger, that it yielded like a piece of thin pasteboard. Dr. W. attributing the death to *Laryngeal Asthma,* did not think much of the case, till about a twelvemonth afterwards, when he happened to meet with, in the writings of M. Elsaesser on *Atrophy of the cranium,* an exact description of its leading features. According to this author's observations, the occipital bone, in this morbid state, will often be found on dissection to exhibit several solutions of its osseous continuity, the vacant spaces being then filled up with nothing but membrane. The periosteum is usually highly vascular, thick, and firmly adherent. Such a state of the cranial bones M. Elsaesser regards as the commencement of rickets; this disease subsequently affecting other bones of the skeleton, if the patient survives. As a matter of course, the children affected with it are always poor weak ailing creatures, with big heads, pale bloodless complexions, and tumid bellies. These are the usual victims of that disease which, under the names of *thymic asthma, laryngismus stridulus, laryngeal asthma,* &c. has attracted so much notice of late years. M. Elsaesser prefers to call it *tetanus apnoieus* (*i, e.* non respirabilis) *periodicus,* and attributes it to a transitory congestion of the brain, which has become the more readily excited in consequence of the attenuation of the cranial bones. The disease most frequently occurs in the second trimestral period of life. A common symptom in such children is, that they are fretful and uneasy, whenever they are laid down; while they may generally be pacified by taking them up in the arms, and keeping the head well supported.

Dr. Widtmann relates no fewer than *nine* cases of this infantile asthma, in every one of which, he assures us, there was a very obvious and easily recognisable softening of the occipital bone. In one, which proved fatal, this bone is described as being externally of a deep blue colour, and as thin and yielding as a piece of parchment; the diploe was soft and full of a sanguinolent fluid.—*Medicinisches Correspondenzblatt Bayerischer Aertze.*

---

### On Intra-thoracic Solid Tumours.

The history of these morbid growths has hitherto attracted very little notice from any pathological writer. Dr. Gintrac of Bourdeaux, twenty years ago, published a memoir on the diagnosis of various thoracic affections. Among these he mentions several instances of Steatomatous and Tuberculous tumours connected with the pleuræ; and now his son M. Henri Gintrac, following the footsteps of his father, has, in his recently published "Essai sur les Tumeurs Solides Intra-thoraciques, 1845," collected together the histories of thirty-two cases of solid tumours—developed within the chest, but not appertaining to, or primarily connected with, the thoracic viscera. The structure of these growths or tumours was, as might be expected, very different in different cases: sometimes it was *encephaloid*, at other times *scirrhous*, and in a third set of cases it was *tuberculous*. In several instances it has been said to be of a *steatomatous* nature. We shall briefly relate a few cases of this rare affection.

*Case.*—A man, 75 years of age, had been for a length of time asthmatic, and much distressed with palpitation of the heart upon any exertion. He became dropsical, and gradually sunk and died.

On dissection, a circular, somewhat flattened, osseous tumour was found at the lower and inner part of the right lung, the substance of which remained perfectly sound, but somewhat compressed. The left lung also appeared quite healthy; but it had been pushed upwards and backwards against the vertebræ, the lower part of the pleural cavity being entirely occupied with a large tumour that had become developed there. It proved to be a cyst with hard bony parietes, adhering by its upper surface to the substance of the lung, and by its lower to the diaphragm. The question naturally suggests itself, how are we to account for the development of such tumours in the cavity of the chest? If we attempted an answer, we might be inclined to say—perhaps by the osseous transformation of false membranes that had been formed during an attack of pleuritis at some former period. Several cases are recorded by M. Gintrac that were analogous to the one now briefly related; and in the history of one or two, it is not difficult to follow the connection of the phenomena which seemed to trace back the formation of the intra-thoracic tumour to an old pleuritic attack.

In other cases, the development of the morbid growth had clearly nothing to do with any previous inflammation in the part; for it was of an encephaloid or scirrhous nature.

The situation of such tumours was sometimes between the pleura costalis and pleura pulmonalis; at other times, it was between the latter membrane and the substance of the lungs; or between the former and the ribs. Occasionally they occupied the place of the mediastina; and in a few rare cases they seemed to have originated in the osseous substance of the ribs themselves. Some of the cases of genuine intra-thoracic tumour have, at different times, been published as examples of degeneration or of hypertrophe of the Thymus gland—an opinion which M. Gintrac considers to be quite erroneous.

It is rare that these tumours have been diagnosticated during the life of the patient. Occasionally indeed this has been the case, as in the following instance.

A man, who had for many years been subject to catarrhal complaints in the winter season, had an attack of pneumonia in 1830. In the end of May 1839, he received a heavy blow on the lower and anterior part of the chest. On the following day, he found two distinct swellings in the part that had been struck. Being examined at St. Andrew's hospital in Bourdeaux, it was found that one of these swellings rested outwardly upon the cartilages of the third, fourth, and fifth ribs, and inwardly upon the corresponding part of the fore surface of the sternum. It was irregular and knobby on the surface, resisting to the finger, without any sense of fluctuation, and firmly adherent to the subjacent parts. The second swelling, not more than four *centimetres* apart from the other, was larger, and of a hemispheric shape. It rested on the cartilages and anterior extremities of the eighth, ninth, tenth and eleventh right ribs, being situated externally, or rather farther back from the median line than the other; it was equally unyielding and immoveable.

The patient was suffering a good deal, at the time of his admission into the hospital, with dyspnœa: this became gradually worse and worse, and he died fifteen days afterwards.

From the external appearances, coupled with the existence of symptoms that indicated a compression of the lungs, M. Gintrac senior gave it, as his opinion, that there was an intra-thoracic tumour in this case.

On *dissection*, a large tumour was found to occupy the entire front mediastinum, adhering firmly to the posterior surface of the sternum, and the cartilages of the ribs. The heart was pushed back, and the lungs compressed against the vertebræ. Similar morbid growths were found attached to the right clavicle, and to the inner surface of the 10th and 11th ribs on the right side.

Now, even in this case, it is very doubtful whether any correct diagnosis could have been formed, if it had not been that the presence of the extra-thoracic tumour naturally enough suggested the idea of the co-existence of similar growths within the chest.

In a somewhat analogous case, MM. Corvisart and Leroux mistook an immense fibrous tumour, compressing the left lung, for an effusion of fluid into the pleura. The case is altogether so interesting, and represents so well the phenomena which usually accompany the existence of tumours within the cavity of the chest, that we are induced to give the particulars of it.

A man about 33 years of age, accustomed to hard labour, was subject to alternations of profuse perspirations and sudden chills; he was attacked with cough, accompanied with mucous expectoration; to which were soon added hoarseness and dyspnœa, along with a sensation of pricking and pain, extending from the throat to the extremity of the sternum, and which was sometimes so acute as to occasion fainting. The dyspnœa increased daily. Some months after, hemoptysis supervened; the pains augmented; the beatings of the heart became more frequent and tumultuous. The lower extremities became œdematous, and the sleep uneasy and agitated. When he entered the hospital, his face was pale and puffy, and his eyelids were œdematous. A slight pain was felt towards the larynx and at the commencement of the trachea; the sputa were puriform, often tinged with blood; cough frequent; respiration short, deep and sighing. The left side of the chest was more *bombé*, and rounded than usual; on percussion it did not give out any sound. The right side gave an obscure sound anteriorly, and a more clear one on the back and side. On applying the hand over the heart, no movement or pulsation of that organ could be felt. The patient preferred the sitting posture, inclining forward; but he could lie down either on the back or on each side, though more frequently and with greater ease upon the left one The sleep was troubled with painful dreams. The pulse in both arms was small frequent, and tolerably regular; generally, a little less strong on the left than on the right side. The skin was slightly œdematous over the whole surface of the body: the feet were much engorged.

Corvisart, judging as much from the symptoms manifested since the attack of the disease, as from the signs furnished by the actual inspection of the patient, having especial regard to the total absence of sound in the left side of the chest, thought it was a case of effusion, filling all the left cavity of the thorax, and compressing the lungs so as to annihilate their functions. The breathing became worse, the expectoration altogether purulent, and the patient gradually sank and died.

*Dissection.*—In place of the effusion that had been predicted, there was a solid mass, of a reddish white colour, and of an irregular and nodulated surface, which filled the left side of the thorax, occupying also the usual place of the mediastinum, and extending upwards and in front on the right side. The left lung, of which the parenchyma was almost entirely disorganized, was reduced to a state that was almost lamellar: it contained besides an abscess in its substance. The right lung was considerably diminished in size; and the mediastinum, the pericardium, and the heart were pushed into the right cavity. The tumour was evidently situated between the debris of the upper lobe of the left lung, and the mediastinal pleura.

Pleural effusion is not the only morbid condition that may be mistaken for the presence of intra-thoracic tumours. M. Gintrac relates several cases where these growths have been accompanied with all the usual symptoms either of pulmonary or of cardiac disease. In a few instances, the most conspicuous symptoms were those indicative of hepatic derangement. With all our improved means of diagnosis, derived from Auscultation and Percussion, the case will almost always be one of perplexity and doubt. If there happen to be any outward tumours on or near to the thorax, and if the constitution of the patient be decidedly cachectic, and especially if the shape of the chest has become visibly altered since the first occurrence of the symptoms, we may possibly be led to form a correct opinion. The age of the patient may partly assist us; for most of the cases, hitherto observed, have occurred in middle-aged and old persons. In more than one instance, the aggravation, if not the invasion, of the symptoms was traceable to a blow or other injury of the chest. In almost all the cases collected together by our author, the heart was observed to be more or less displaced from its natural locality: this therefore is a phenomenon that deserves to be taken into account in our attempts to form a diagnosis. As a matter of course, the symptoms must vary alike in their character and intensity, according to the parts which are compressed or otherwise injured by the unnatural production within the thoracic cavity.

---

### Singular Case of rapidly-fatal Emphysema: pultaceous Softening and sub-peritoneal Rupture of thr Stomach.

The editor of the French Medical Gazette remarks that the following is one of the most remarkable cases on record. It certainly does seem very strange that Emphysema, proving so rapidly fatal, should have proceeded from a lesion of the stomach, or indeed of any other organ save the lungs. It is much to be regretted that the report of the case, more especially of the *post-mortem* appearances, is so incomplete.

A medical gentleman was suddenly taken ill immediately after eating. His breathing became laborious, and his voice scarcely to be heard; the skin was chilly and anserine, and the pulse small and contracted: the abdomen at the same time was exceedingly distended. No sooner was an enema administered, and the patient had begun to strain in the act of defæcation, than his neck was observed to become emphysematous, and he was threatened with suffocative dyspnœa. The action of the lungs seemed to cease; his face assumed a purplish hue; the puffy swelling increased at every inspiration, during every act of which the patient appeared to be making, in spite of himself, the movements of swallowing. In the course of a very short time, his appearance was scarcely that of a human being, so swollen and puffed out was every part of the body; but, from time to time, there issued a moan that indicated the inward sufferings of the patient. A burning thirst made him continually ask for drink; but the greatest difficulty was evidently experienced in swallowing. He expired in the course of a few minutes, immediately after an effort to swallow a mouthful of water: the suffocation was complete. The corpse was soon excessively distended, so that it looked more like a blown-out bag than a human body.

*Dissection.*—The abdominal parietes, enormously expanded, did not sink down when an incision was made into the cavity of the belly, which did not contain any gas. Upon a transverse incision being made, there was exposed the stomach: this viscus was so distended, that it protruded through the wound, and was found to extend from the epigastric region into the iliac fossa: its muscular coat seemed to be hypertrophied. On drawing it forwards, the epiploic fold attaching it to the diaphragm was torn across; and then it was discovered that air escaped from a large aperture in it, extending from the cardiac to the pyloric orifice along its small curvature. The substance of the parietes of the stomach along this extent had undergone a pultaceous softening: the mucous coat was highly injected with dark-coloured blood, and appeared to become sensibly more and more attenuated in approaching the seat of the ramollissement. The *lungs* were squeezed up against the vertebral column: the *heart* was full of dark blood. There was no serious lesion anywhere, save and except in the stomach, as we have just described.

Professor Burgraeve, who has given the preceding (very imperfect) history of the case, explains the suddenness of the fatal event in the following manner: "A rupture of the stomach must have taken place under the peritoneum, and the air, forcibly drawn in by the movements of the chest into the vacuum that was produced, have become extravasated into the general cellular tissue, chiefly along the vertebral column where this tissue is loosest, and whence it was diffused to every part of the body. Hence those violent efforts of deglutition that were so marked during life. The thoracic viscera and the great blood-vessels must have first experienced the effects of the compression, especially from the column of air ascending along the posterior mediastinum. In the neck, the pressure must have been greatest, in consequence of the aponeurotic bands between which are situated the trachea and the vascular trunks, arterial as well as venous. The extravasated air had stopped at the cranial vault, none of its apertures having afforded an egress to it, and had left the brain intact. This organ had not suffered until subsequently, by the unceasing progress of the asphyxia." —*Annales de la Societé de Med. de Gand.*

---

### On the Use of Ioduret of Potassium in Syphilitic Affections.

The report, which M. *Gauthier* has recently published respecting the curative power of this salt of Iodine in secondary and tertiary syphilitic affections, is on the whole highly favourable to its use. He has administered it in a vast number of cases, and has rarely noticed any injurious or even unpleasant effects fairly attributable to its operation. On a few occasions it appeared to cause a salivation; which, however, speedily ceased. Now and then, an innocuous exanthem made its appearance on the surface. In some persons it causes slight gastric irritation; but in most, the digestive functions appear to be decidedly improved under its use. In no instance has any wasting of the body seemed to be induced by it, as has occasionally been observed with respect to Iodine. One of the most constant effects of the Ioduret is to increase the flow of the urine. It seems to pass very rapidly into this and the other secretions; its presence is readily discoverable by its well-known appropriate tests. M. Gauthier has often detected it in the saliva.

The following are the forms of the syphilitic disease in which he has witnessed the most decided curative effects. Pains of the *bones*, even when most severe, are often very rapidly and effectually subdued; nay, when caries exists, a salutary change is not unfrequently obtained. Thus in Ozœna, complicated with disease of the palate or nasal bones, we seldom fail in greatly benefiting, if not in curing, the disease. In various tubercular affections of the *skin* and *mucous membranes*, the Ioduret will be found most useful. Deep ulcerations of the throat and pharynx, rhagades or fissures about the anus and nails, will not unfrequently heal up most satisfactorily, even when mercury has been previously tried and failed. It is sometimes truly marvellous to witness the decided improvement of the general health in the course of a few days, under the use of the Ioduret when judiciously administered. M. Gauthier considers that it is a most valuable remedy in many cases of mercurial cachexy: an ioduretted gargle will often serve to check salivation from this cause.

He invariably begins its administration in small doses—from two to four grains, or even less, twice a day. The quantity should be doubled every third or fourth day, until it reaches 15 or 20 grains. This dose should be continued for some time; but, if it fails in producing any decided effect upon the disease, it may be increased to two scruples or even a drachm. In a few cases, he has given as much as two drachms in the course of the twenty-four hours.

A solution of the Ioduret in water, to which some tincture of Iodine has been added, may be advantageously used as a gargle in ulcerated sore-throat, and as a wash to ulcers on the surface, or on the Schneiderian membrane.

The average period, during which the internal use of the Ioduret should be continued, may be stated to be from six to eight weeks. Much will depend on the gradual increase of the doses given. Many cases will remain stationary, if the quantity of the salt administered be not progressively—and this, too, rapidly—augmented.—*Observations pratiques sur le Traitement des Maladies Syphilitiques par l'Iodure de Potassium, by M. L. Gauthier. Paris,* 1845.

---

### Isopathia, or the Parallelism of Diseases.

Dr. Harden of Georgia, U. S., applies the term *Isopathia** to express that law or

* " ισον παθος, æqua affectio. The term *isomerism*, in chemistry, while it furnishes an analogy in etymology, affords, also, a good illustration of our use of the above. It is known that there are many inorganic bodies with different external appearance, which consist of the same elementary parts, while others, essentially distinct in composition, present the same external form. To these last the term isomorphic has been applied; and even here the analogy holds in disease, for, as we have seen, there are many so-called diseases, with the same external form or type, so far as regards their seats and symptoms, which are still pathologically distinct. To such diseases the term *isotypic* might be very properly applied. Diseases, then, may be *isopathic* and *heterotypic*, or *isotypic* and *heteropathic*, as we hope to make appear in the course of our remarks."

general feature of disease, whereby "many symptoms and groupes of symptoms, which have been and still are regarded as distinct diseases, and arranged as such by systematic writers, may be referred to the same pathological state of the system, and may mutually replace one another in the same or in different individuals."

The disposition of diseases to wear each other's livery has long been remarked by some of the most eminent physicians. Sydenham has expressly declared that "epidemics of continued fevers, although *resembling each other in respect to many of their symptoms and general characters, are often very different from each other;* for a mode of treatment which is useful in one, may be decidedly pernicious in another." Hence the importance which he has attached to the epidemic constitution of the air, in determining the true character of these diseases. Hunter declared that "diseases of the *same specific nature* not *only vary in their visible symptoms or actions,* but in many of *those that are visible,* arising, probably, from peculiarity of constitution and causes, which will make the effects of application vary almost in the same proportion." Dr. Alison says, "when changes in the structure of the body, that take place in the course of disease, are examined during life, and more especially after death, it appears that *different combinations of symptoms are often apparently excited by the same fundamental diseased action, and, consequently, found in connection with the same alteration of organic structure;* and again, that very *different alterations of structure may be attended, in different individuals, by symptoms, the greater number of which are very nearly the same:*"—and Andral, in his recent work on Pathological Hæmatology, has advanced the same sentiment: "These various facts," says he, "are not inconsistent with one another; they only throw more light on one of *the most important of medical truths,* namely, that two *diseases may have identical symptoms without being of the same nature,* and that however close their resemblance, they may still require different modes of treatment, because very different conditions of the economy may give rise to or maintain them."

Dr. Harden very justly remarks that it has been, in a great measure, from the ignorance or neglect of this important physiological law, that so much discrepancy has existed among medical men as to the true nature and the proper treatment of the *same supposed disease,* as well as the varying success of the *same remedies in the same supposed cases.*

With the view of giving greater consistency and perspicuousness to his illustrations of the Isopathic law, he divides diseases into the following *generic* types, under which he proposes to treat of the individual species and their modifications.—1, Febrile types; 2, Inflammatory types; 3, Purulent types; 4, Tuberculous or Strumous types; 5, Scorbutic or Hæmorrhagic types; 6, Exanthematous types; 7, Hydropic types, and 8, Gouty or Podagric types.

In the present article, he confines his remarks to the class of Pyrexiæ, or Febrile diseases, which, we need scarcely say, afford numerous instances of isopathic connection. It must be confessed that the pathological history of this most common groupe is still very imperfectly understood. The discrepancy among authors in regard to the (alleged) seat of the same fever is sufficient evidence that this disease has no determinate or invariable locality nor primary "point de depart" in the system.

"If we were to judge," says Andral, "by post-mortem examinations only, we should often believe that inflammatory, bilious, catarrhal or mucous, typhoid or adynamic, nervous or ataxic, fevers, were *one and the same disease.*" It would be easy to quote a host of authorities to the same effect; but space will not permit this at present.

Fevers have been variously divided and arranged by different authors. The most generally adopted division has been into Intermittent, Remittent, and Continued. But there are several objections of great weight to this arrangement. For example, it is now pretty generally admitted that Intermittent and Remittent fevers are only different *types* of the same disease. Many of the most able and practical writers within the last ten years have, in the most distinct and unqualified language, advocated this position. We shall select only one example. M. Maillot, the chief physician of the French Military Hospital at Bon in Africa, in treating of the fevers of Algiers, says: "Whoever will consider the facts now stated cannot hesitate to admit that the Remittent and Continued fevers of the hot season, in such a climate as that of the Northern Coast of Africa, are in truth Intermittent fevers, complicated with some local mischief, which has the effect sometimes of merely obscuring the features of the original disease, and at other times of masking them altogether, so that they cannot be recognised;" and again, in speaking of the treatment to be pursued, he uses the following emphatic language:—"According to the opinion which we form of these Intermittent, Remittent, and Continued Fevers—which alternately succeed and replace each other, at one time disappearing, to break out at some future period,—and the connection which we may recognise to exist among them, however different they may often seem to be in their symptoms, will depend the choice of either a sound or a fallacious mode of treatment."

Dr. Rush was of opinion that the Tertian is the original type, and that the double tertian is really and truly the form in which all our (American) autumnal remittents present themselves, and that the difference in the intermissions depends upon the lengthening or shortening of the paroxysms, so that they either run into each other or have a complete interval between them.

By examining the successive periods or stages of a febrile attack, we shall be better enabled to point out various features of Isopathic connection. The degree of *coldness*, with which it is usually ushered in, varies exceedingly—from a mere slight sensation or "aura," in perhaps only one part of the body, to a universal shivering, and chill of the whole surface perceptible by another person. Now in some cases this may be the only symptom of the disease that is manifested—a slight chill that passes away, without being succeeded by other symptoms. Most medical men would probably regard such a phenomenon as nothing more than a slight hysterical feeling. The "aura epileptica" seems to be something of this kind.

The cold fit of an Intermittent may shew itself by a torpor or oppression of the nervous system—varying in degree from a simple *drowsiness* to a state of complete *coma* or *apoplexy*. Fevers, associated with soporose, apoplectic and paralytic symptoms, have been described by writers of almost every country. Sydenham, Werlhoff and Morton appear to have been the first who regarded them as modifications of intermittents. "Some years ago, autumnal fever appeared in the interior of the southern states (America) in this form, and was in many places denominated the *cold plague*." The Italian physicians have repeatedly described this form of the disease. This apoplectiform seizure may constitute the whole apparent disease; and, if no time be given for the development of its true character, the case might be treated as one of Apoplexy, and the patient die under the treatment that would most probably be pursued. Andral has detailed the case of a patient who exhibited all the phenomena of "intermittent apoplexy," which in its turn observed the same regularity as the fevers of this name. The quinine was administered, and the individual quickly recovered.

The apoplexy may be replaced by some form of Paralysis—as hemiplegia, paraplegia, or local palsy. Dr. Macculloch has treated this subject at considerable length in his most interesting work on Remittent and Intermittent Diseases, and we cannot do better than refer the reader to it for information.

*Painful or Neuralgic Symptoms.*—The most usual seat of pain, in the early or cold stage of a febrile attack, is the back and head; and very often this pain is evidently of a neuralgic character. The alternation of the pain in the back with gastralgia, headache, or even with common neuralgia of the face, is a frequent occurrence: we may therefore very reasonably suppose that the former is owing to some affection of the spinal nerves. In some cases, the pain is confined to one part: of this nature is the lumbago, sciatica, or hemicrania, that is often present. It is quite unnecessary to adduce authorities to prove that these and other forms of neuralgic suffering are very often connected with, if they do not actually arise from, the morbific cause of intermittent fevers, viz. malaria.

*Convulsive or Spasmodic Symptoms.*—The rigor, with which Fevers usually set in, may be considered as of this character. The convulsive tremor may be either very general or local, either of a tonic or of a clonic character. The rigor is sometimes so indistinct as to be entirely overlooked; or it may be replaced by other symptoms, such as twitchings of the muscles in various parts of the body. These twitchings or spasms may be merely temporary and pass away without leaving any more serious disease; or they may become permanent, giving rise to such a muscular contraction as we see in Wryneck, Club-foot, &c. The "globus hystericus" is perhaps a symptom of a somewhat similar character. In some cases, a spasmodic Cough—probably connected with an irregular action of the diaphragm—has seemed to take the place of the rigor; for the accession of the febrile fit has usually set in with this symptom. In other cases, Asthma has been the substitute. The periodicity of the attack of the dyspnœa, and its arrest by the use of quinine, naturally lead to this conclusion. Dr. Macculloch is of opinion that one form of Pertussis may be symptomatic of marsh fever. Palpitation of the heart likewise may be of this character; and so may Hiccup. Pleurodyny, or stitch in the side, is unquestionably of an intermittent character in some cases. Spasm of the intercostal muscles is a very common affection among the Negros in America: the pain in the side being the chief and most distressing symptom, while the febrile excitement may be obscure and imperfect.

By far the most alarming of the substitutional maladies that are apt to occupy the place of the cold stage of a remittent or intermittent, and are attributable to the same malarious origin, is Tetanus. Dr. Rush has expressly affirmed "that yellow fever sometimes puts on the form of tetanus." One of the most interesting examples is that published some years ago by M. Dance, and in which the tetanus assumed the intermittent type, and the accompanying fever exhibited sometimes a remittent and at other times a distinctly intermittent character.

Colic and Vomiting, in certain localities and epidemics, are not unfrequently observed in lieu of the regular shivering of the first stage of a febrile paroxysm. Dr. Macculloch remarks that the latter (vomiting) sometimes "becomes the most conspicuous symptom, or even the only one which the patient may notice; yet inattentive practitioners are subject to mistake it for an original disease, dependant upon some mysterious cause, or to assign a wrong one for it."

In children, Convulsions so frequently occupy the place of the rigor of a febrile attack, that Dr. Harden does not hesitate to lay it down as a rule that *the rigor of an adult is the convulsion of a child in our climate fevers.* There is reason for believing that even in adults the rigor may sometimes assume the violent form of an Epileptic paroxysm; and it appears not improbable that some of the forms of Hysteria might be robbed of half their mysterious character, by studying them in connection with intermittent fevers. Dr. H. suggests that Laryngismus Stridulus also may occasionally be a modification of the rigor of an agueish fever.—*The American Journal of the Medical Sciences,* No. XVI.

(A very valuable paper on an interesting subject. We trust that Dr. Harden will follow it out.)

---

### On the Nature of the Black Vomit in Yellow Fever.

Dr. Nott of Mobile—not far from New Orleans—has communicated an instructive paper, to the American Journal of the Medical Sciences for April 1845, on the " Pathology of the Yellow Fever," as observed by him in that town and district. He regards this most formidable disease as produced by the introduction into the system of a morbific miasm, the effect of which is to vitiate and corrupt all the fluids of the body. He is therefore a decided Humorist in his pathological views, utterly rejecting and emphatically denouncing the pernicious doctrine of Broussais and his followers, who would make but little difference between the Pyrexiæ and the Phlegmasiæ. " It has become"—he says with great force and truth—" the fashion to speak of yellow fever becoming ' *localized;*' but to my mind it is just as rational to say, that a storm has ' *localized itself,*' because it blows down a tree here and there, whilst it is shaking the whole forest to its foundations. That the yellow fever does, in the course of its progress, like all great epidemics, give rise to morbid changes in particular organs, no man of sense will deny; such effects are to be expected from all poisons which pass into the blood, or act in any way on the system. Sometimes in this disease there is gastritis; the liver is often changed in a remarkable degree; the kidneys are found altered in colour and texture; the lungs show dark patches; the brain is congested; bloody tumours form; the secretions are checked; the texture of the skin so changed that I have seen (as in the case of Capt. Atwood, last summer,) the cuticle, in attempting to cleanse the face with a wet towel, wiped off, and the skin left as raw as a blistered surface, and this too twelve hours before death." All that we propose at present is to give Dr. Nott's views on the origin and nature of the " black vomit." The following extract will best convey his sentiments on this subject.

" With the assistance of my friend, Dr. P. H. Lewis, I have tested the black vomit in a considerable number of cases this summer (1844), and in every instance I have found it to be acid; when ejected from the stomach during life, it invariably turned litmus paper red, and the aqueous portion of that, which was taken from the stomach after death and filtered, in several cases effervesced strongly with carbonates. The aqueous portion, thus filtered, differed in colour; in some it was perfectly limpid like water; in one, of a light green colour like dilute bile with an acid added; and in others, it was of a deep brandy or rum colour; which appearance was no doubt given by a small admixture of blood.

" The secretions of the stomach in yellow fever are often excessively irritating, and this property is probably attributable to the presence of acid; the patient often complains in the black vomit stage of a burning or scalding sensation in the stomach, which is immediately relieved by throwing off its contents. The patient, too, often complains of the black vomit scalding the œsophagus, which, after death, is usually found more or less denuded of its epithelium. The acidity of this secretion may possibly account for many of the morbid changes in the stomach and œsophagus. A morbid secretion of tears will scald the cheek; mucus from the nose inflame the lip; morbid secretions from the bowels excoriate the anus; morbid bile irritates the stomach and bowels, &c.—and we know that the gastric juice will often corrode the stomach in a short time after the extinction of life.

" The next step was to ascertain whether acids would with blood produce a compound with the characters of black vomit. I accordingly took a few drachms of blood from the heart of a patient dead of yellow fever, and added to it four or five drops of muriatic acid, diluted with a drachm or two of water, and shook them well together; the black colour was produced instantly. The same experiment was tried repeatedly on the blood of yellow fever patients, and on that drawn from a patient with pleurisy by cupping, and the effect was invariably the same.

" Any one wishing to form a correct idea of black vomit, has only to treat blood in this way, and add a little gum-water or flax-seed tea to represent the mucus of the stomach, and his curiosity will be gratified; no one can tell the artificial from the genuine black vomit."

With respect to *treatment*, Dr. Nott very justly remarks that, what may be proper and salutary in one Epidemic of the Fever, may prove most pernicious in another. " He, who is ignorant of the various types, in which this Proteian disease appears in different years and in different latitudes, must either not have read, or read to little purpose, the history of Yellow Fever. In one epidemic we are told that the lancet is the sheet-anchor; in another, it is death. This dfference occurs to a limited extent in Mobile; but the rule is, *beware of the lancet*."—*Ibid*. No. XVIII.

---

### ON THE DEPOSITS OF CARBON IN THE SUBSTANCE OF THE LUNGS.

The following remarks are intended to illustrate the nature of that black matter that is met with, almost constantly, in the respiratory organs of old persons. All the cases that have been examined by me, says Dr. Guillot, physician of the Hospice de la Vieillesse at Paris, have occurred in men above 70 years of age; and in not a single instance had the patient followed any trade, the pursuit of which necessarily exposed him to the inhalation of carbonaceous particles. Most of my patients had been coachmen, gardeners, bakers and so forth. Although, as might be expected at such an advanced period of life, one or more of the viscera were generally found on dissection to be diseased, the cause of death in the majority of cases was unquestionably due to the more or less rapid and complete interruption of the aerial or sanguineous circulation of the lungs, in consequence of the carbonaceous deposit in the pulmonary parenchyma. It has only been since the beginning of the present century that the attention of pathologists has been specially directed to the examination of those black deposits, that are so frequently met with in this structure.

Various opinions have been entertained by different pathologists as to their nature. Bichat* regarded them as minute bronchial glands; Breschet,† as formed by the exhalation of the blood into the fatty cellules or utricles; Heusinger,‡ as deposits of a carbonaceous pigmentary substance, the result of the imperfect oxydation and decarbonisation of the blood; Trousseau,§ as produced by cruoric globules. According to the latter gentleman and M. Andral,|| the deposition is the result of a secretion; it is regarded by Andral and Grisolle¶ as connected with the existence of chronic pneumonia; while Laennec**, Gregory†† and others have traced it to the inhalation of sooty and carbonaceous particles from a smoky atmosphere. Berard‡‡ says that " the black matter of the lungs, which is not blanched either by chlorine or by nitric acid, owes its dark colour to carbon;" and more recently M. Bourgery§§ alludes to it as " a veritable deposit, apparently

---

* Traité d'Anatomie Descriptive, t. iv. p. 22, Ed. 1839.

† Considerations sur une Alteration Organique appelée Degenescence noire, Melanose, &c., 1821.

‡ Recherches sur la Production Accidentelle de Pigment et de Carbon dans le Corps Humain, &c. Eisenach, 1823.

§ Archives Generales de Medecine, t. xvii., 1828.

|| Precis d'Anatomie Pathologique, t. i. p. 459.

¶ Traité Pratique de la Pneumonie à ses differents Ages.

** Traité de l'Auscultation, t. ii. p. 312, et seq.

†† Ed. Med. and Surg. Journal, 1831.

‡‡ Texture et Developpement des Poumons, 1836.

§§ Notice sur les Titres de M. Bourgery, 1845.

carbonaceous, and analogous to the deposit that lines the chimneys of our fire-places."

Dr. Guillot is of opinion that every one of these opinions is more or less faulty, and he undertakes to prove—

1. That the black matter, so often found in the lungs of old persons, is not formed by the blood or by cruoric particles, as Breschet and Trousseau have supposed;—neither is the result of any process of secretion, according to the opinion of Andral—nor has any analogy with the pigment of the skin or choroid coat—nor yet is owing to the inhalation of a smoky atmosphere.

2. That it is really and truly of a carbonaceous nature; but that the carbon is not derived from the process of chemical re-agents, but is deposited "en nature," during the continuance of human life, in the parenchyma of the respiratory organs.

3. That the presence of this carbon, by its gradual increase, may occasion—chiefly in the more advanced periods of life—certain morbid phenomena that are appreciable by the physician.

4. That the accumulation of this matter may be so extensive in the lungs as to cause death, by impeding the circulation and respiration; and that, in many cases of acute or chronic pulmonary disease in old people, the presence of these carbonaceous deposits adds considerably to the danger, and may account for the frequent fatality, of the pneumonic attack.

Dr. Guillot adds that these carbonaceous deposits appear to have considerable influence on the various modifications which tubercles undergo in certain constitutions: "the majority of patients," says he, "in whom the progress of Phthisis has been modified or arrested, exhibit in their lungs on dissection, if they have reached an advanced period of life, a more or less extensive deposit of carbonaceous molecules."

Dr. G. has given the particulars of some chemical experiments, performed by M. Melsens, to ascertain the nature of melanotic matter obtained from the lungs. The result of them was clearly to shew that it is of a truly carbonaceous nature.* On one occasion Dr. G. found, in the lungs of an old man, a compact mass of carbonaceous matter arranged in layers; it was black, very hard, having a shining metallic fracture, infusible, burning on a platinum plate without flame, and without giving out almost any smell. This matter, when burnt in a stream of oxygen gas, yielded nothing but water and carbonic acid.

The state of extreme division of this carbonaceous matter, as it is obtained by treating the lungs successively with acids, alkalis, water, alcohol and æther, enables us in a measure to form an idea of the hardness which in some cases it may acquire. It has been known to exhibit a brilliant aspect and the metallic lustre of the carbon, that is obtained from the decomposition of spirit of turpentine in a porcelain tube heated to redness.

We have already said that the deposit of melanotic matter in the lungs is almost peculiar to old age. Certain it is that it is rarely or never met with in infancy and youth; no appearance of it being usually discoverable until about the period of middle life. At first it looks (we require the microscope for this purpose) like a very fine black powder, sprinkled through the transparent substance of the pulmonary tissue. This powder is found to be constituted by the assemblage of excessively minute granules, separated more or less completely from each other; the intervals between them becoming less and less, as the amount of the deposit increases. The molecules seem to be quite impervious to the light; for, even under a high magnifying power, they look as intensely black as when seen with the naked eye.

---

* *Recherches Chimiques sur la Matiere des Melanoses;* Compte Rendu des Seances de l'Academie des Sciences de Paris, t. xix. p. 1292.

The extent, which these molecular deposits may occupy in the centre or on the surface of the lungs, is sometimes immense. In some cases, the pulmonary tissue is so deeply stained, that we can readily observe the accumulation of the black matter with, or even without, the aid of a lens, by placing a portion of the lung in pure water. Dr. G. is of opinion that the seat of the carbonaceous deposit is in the intervesicular or inter-canalicular spaces of this tissue, and not on the mucous surface of the extreme air-tubes. At this early stage, there is usually no other morbid alteration or abnormal condition of the pulmonary tissue discoverable. But when the quantity of the deposit is considerably increased, certain modifications, either of the air-tubes or of the minute blood-vessels, or of both, are usually observed to have taken place. These modifications are obviously the result of compression upon these parts, in consequence of the accumulation of foreign matter in their neighbourhood: the air-vesicles or tubules are obstructed, and the blood-vessels are rendered impervious. It is usually in the upper lobes of the lungs that the amount of carbonaceous deposits is most considerable: there is always a less amount in the lower, than in the upper and middle, lobes. When there is a considerable deposit near the pleural surface of the lung, there is very generally the appearance of a dark-coloured cicatrix at this point—a feature that has often been mistaken for the trace left by a cicatrised vomica. It arises from the solidification of the pulmonary tissue, in consequence of the accumulation of carbonaceous matter underneath.

The melanotic deposit may be either diffused in small molecules over a wide extent of the lungs; or it may be accumulated in nuclei or masses, varying in size from that of a hemp-seed to that of a walnut or even of an orange. Dr. G. gives the following description of the appearances which he has sometimes met with on dissection, when the accumulation has been considerable.

"The rest of the pulmonary substance, deprived of its air-tubes and blood-vessels, forms then a sort of *gangue* which resists the edge of the knife, is sometimes very much indurated, inelastic, not unlike a piece of moistened pasteboard that has been stained dark, or to leather that has been boiled in water loaded with smoke-black. These very abnormal matters, in the midst of which the carbon is contained, do not lose their dark colour upon washing; they putrefy very slowly; and even in the act of decomposition, they continue to retain the black stuff with which they are penetrated. It thus appears that the charcoal is not to be separated by washing. Whatever be the force of the stream of water used, or the degree of putrid dissociation of the elements of the affected tissues, it requires a still more powerful action—one that is capable of entirely destroying their organisation, without acting upon the carbon—to enable us to procure it by itself."

Occasionally we find that, in the centre of one of these masses of carbonaceous matter, there is a space occupied with black matter that is in a diffluent state, and which may be washed out with a stream of water, so that there is left behind a cavity proportionate to the extent of the *ramollissement*. When this dark fluid is examined with the microscope, it is found to be loaded with carbonaceous particles, which exhibit the same chemical characters as the ordinary melanotic deposits do.

Dr. G. says that he has never seen a case wherein the melanotic matter was truly encysted, or continued in a distinct cyst or sac. This peculiar feature, and the circumstance too of the matter always having a molecular composition, may serve to distinguish this morbid formation from others of a similar appearance, occasionally found in the brain, liver, kidneys, &c. From repeated most careful examinations with the microscope, he is quite satisfied that the carbonaceous deposits have nothing to do, nor are in any degree connected, with the extravasation of blood in any form.—*Archives Generales de Medecine.*

### On the Pellagra, as observed in France.

This disease having of late years prevailed to a considerable extent in the valley of the Gironde, the French minister of public instruction wrote, some time ago, to the Academy to have a report upon certain documents that had been transmitted to him on the subject.

It was in 1829 that Pellagra was first distinctly noticed in the "landes" of that district of France. As seen and described by Dr. Marchand, its most conspicuous feature is a squamous erythema of the exposed parts of the body, more especially of the backs of the hands, returning periodically every year in the spring, and accompanied by a series of constitutional symptoms, the severity of which is always proportionate to the persistence of the disease. The eruption—which may be successively papular, vesicular, and pustular—usually disappears in autumn, leaving patches having a shining aspect like that of a cicatrised burn. The constitutional symptoms appertain chiefly to the digestive and the cerebro-spinal systems. Hence Dr. M. characterises them by the appellation of *gastro-entero-rachialgia.* There is usually redness and a fissured state of the lips and tongue, a scorbutic condition of the gums, ptyalism, anorexia, vomitings and diarrhœa. The nervous symptoms are exceeding weakness, vertigo, languor of the sensual and the intellectual perceptions, sometimes delirium and even insanity,—which generally assumes a form of monomania, leading the unhappy sufferer to commit suicide by drowning. The patient becomes weaker and weaker, occasionally dropsical, and at length dies from exhaustion. Dissection has thrown but little light upon the real pathology of this disease. The essential cause of it seems to be misery and destitution, coupled with exposure to a burning sun and an unhealthy atmosphere. Well and truly may it be designated *mal de misere.* In Lombardy it is sometimes called *alpine scurvy*, and in Spain *mal de la rosa*—from the erythematous redness of the skin, we presume. In France, it has been chiefly observed among the poor that inhabit the districts of Gascony near the shores of the Gulf. The country is most barren and insalubrious, and the people are altogether in a wretched condition.

In endeavouring to find out the probable chief causes of the Pellagra, it is of importance,—says M. Jolly, the reporter of the commission—to bear in mind that the disease is not known to have existed previous to the first half of the last century; and moreover, that it is never observed to exist in many countries where the solar heat is much greater than in those parts of Europe where it has been found to prevail. Still, there are several facts to shew that exposure to the sun's rays has much to do with its production. The simple circumstance of the decline of the disease in autumn, and of its recrudescence in spring, proves this to be the case. It is obvious, however, that, if the skin and general system were in a healthy condition, this effect would not be produced: it is only the tree, that has lost its sap, that is liable to have its bark dried up by the heat of the summer sun. M. Jolly is induced to believe that there is some etiological principle endemic in the localities affected, and inherent in the material life of the country. Everything would seem to point to the nature and productions of the soil, and the quality of the food and drink, as the true sources of this morbific principle.

By a curious coincidence, at the very time that M. Jolly was engaged in preparing his Report on the official documents respecting the Pellagra in the south-western parts of France, M. Roussel had transmitted to the Academy a thesis on the same subject, the chief object of which is to trace out the causes, and the geographical limits of the disease. According to his researches, it would seem that it was not until about the middle of the eighteenth century that the Pellagra made any considerable ravages in Italy, although it had been known for forty or fifty years before. In Spain it seems to have been longer known; whereas, in France, it was not observed until the year 1818. M. Roussel, following out his enquiries, has come to the conclusion that the almost exclusive alimentation of the inhabitants upon Maize, and the deficiency or total want of other more nourishing food, has much to do with the production of the disease.

He adduces various considerations in support of this view; these considerations are derived in part from observing the geographical limits of the Pellagra, and in part also from the chronological history of the culture of the maize. "I have been much struck," he remarks, "with the exact correspondence of historical data on this point with those as to the origin and early development of the disease in different countries.

"It is thus that in Spain, if any doubt exists as to the precise time of the introduction of the maize, there is certainly none that its cultivation in the north of the Peninsula did not become at all important till the seventeenth century. Now, it has been shewn that Spain is the first country where the Pellagra was really known in the first half of the eighteenth century.

"In Italy, the connection between the cultivation of the maize on the one hand, and the appearance of the Pellagra on the other, is established upon sufficient data. It is only since the end of the seventeenth, and especially during the first half of the eighteenth centuries that the cultivation of the maize has gradually prevailed and replaced that of other cereal grains. Now we know that it was towards the year 1750 that the Italian physicians began to meet with the Pellagra.

"In France, although the maize was known in the time of Olivier de Serres, this grain did not acquire any importance till a very recent period; and we have seen that the date of the first case of the disease does not extend further back than 25 or 30 years ago."

"Such are some of the statements of M. Roussel: the subject, it must be confessed, requires much more accurate information than we yet possess, before we can arrive at any definite conclusions. It deserves, however, to be noticed that Maize is, of all cereal grains, that which affords the least azotised aliment, and whose harvests are perhaps the most frequently damaged.

In confirmation of M. Roussel's views, we may state that, so far back as 1798, Thouvenel, in his work on the Climate of Italy, pointed out the striking conformity between the period when the cultivation of the Maize had extended into Southern Italy, and that when the Pellagra had begun to make its appearance there; and, at the late scientific meeting at Milan, Dr. Balardini adduced many potent arguments to prove this very point of enquiry, shewing at the same time that the disease was not at all known in those districts, where the Maize did not constitute the food of the inhabitants. Indeed, so strongly convinced are many Italian physicians of the connection between the origin of the Pellagra and the use of this grain, as the chief article of food, that they have affixed the name of *raphania maiztica* to the disease.—*Gazette Medicale.*

---

### NOTE ON THE ADMINISTRATION OF THE OXYDE OF SILVER.

In our review of Sir James Eyre's "Practical Remarks," we have omitted to state that, it is to Mr. Lane that the profession is mainly indebted for the introduction of the Oxyde of Silver as a useful remedy in various ailments—pyrosis, cardialgia, leucorrhœa, &c. Mr. Lane's first communication upon the subject will be found in the Number of the Medico-Chirurgical Review for July 1840, and well deserves to be generally known.

